# Performance of Narrow Band Imaging (NBI) and Photodynamic Diagnosis (PDD) Fluorescence Imaging Compared to White Light Cystoscopy (WLC) in Detecting Non-Muscle Invasive Bladder Cancer: A Systematic Review and Lesion-Level Diagnostic Meta-Analysis

**DOI:** 10.3390/cancers13174378

**Published:** 2021-08-30

**Authors:** Giorgio I. Russo, Tamir N. Sholklapper, Andrea Cocci, Giuseppe Broggi, Rosario Caltabiano, Angela B. Smith, Yair Lotan, Giuseppe Morgia, Ashish M. Kamat, J. Alfred Witjes, Siamak Daneshmand, Mihir M. Desai, Indebir S. Gill, Giovanni E. Cacciamani

**Affiliations:** 1 Urology Section, Department of Surgery, University of Catania, 95123 Catania, Italy; giuseppe.morgia@unict.it; 2 Department of Urology, Keck School of Medicine, University of Southern California, Los Angeles, CA 90033, USA; Tamir.Sholklapper@med.usc.edu (T.N.S.); daneshma@med.usc.edu (S.D.); mihir.desai@med.usc.edu (M.M.D.); igill@med.usc.edu (I.S.G.); 3 Department of Minimally Invasive and Robotic Urologic Surgery and Kidney Transplantation, University of Florence, 50100 Florence, Italy; andrea.cocci@unifi.it; 4 Department of Medical and Surgical Sciences and Advanced Technologies, G.F. Ingrassia, Anatomic Pathology, University of Catania, 95123 Catania, Italy; giuseppe.broggi@phd.unict.it (G.B.); rosario.caltabiano@unict.it (R.C.); 5 Department of Urology, School of Medicine, University of North Carolina at Chapel Hill, Chapel Hill, NC 27599, USA; angela_smith@med.unc.edu; 6 Department of Urology, University of Texas Southwestern Medical Center, Dallas, TX 75390, USA; yair.lotan@utsouthwestern.edu; 7 Department of Experimental Oncology, Mediterranean Institute of Oncology (IOM), 95029 Catania, Italy; 8 Department of Urology, University of Texas MD Anderson Cancer Center, 1515 Pressler, Unit 1373, Houston, TX 77030, USA; akamat@mdanderson.org; 9 Department of Urology, Radboud University Medical Center, 6500 HB Nijmegen, The Netherlands; Fred.Witjes@radboudumc.nl

**Keywords:** blue light cystoscopy, accuracy meta-analysis, bladder cancer, cystoscopy, narrow band imaging, photodynamic diagnosis fluorescence, PDD, hexaminolevulinate, HAL, 5-aminolaevulinic acid, 5-ALA

## Abstract

**Simple Summary:**

Bladder cancer is one of the most common malignancies in the United States with a majority of patients diagnosed with non-muscle invasive bladder cancer (NMIBC). Despite early detection and regular surveillance of most cases, recurrence and progression rates remain high. The aim of our systematic review and meta-analysis was to compare the sensitivity, specificity, and oncologic outcomes of photodynamic diagnosis (PDD) fluorescence, narrow band imaging (NBI), and conventional white light cystoscopy (WLC) in detecting NMIBC. Through the collection of prospective and randomized controlled trials, we demonstrated that tumor resection with either PDD and NBI exhibited greater diagnostic sensitivity compared to WLC alone. Our findings underscore the value of integrating these enhanced technologies as a part of the standard care for patients with suspected or confirmed NMIBC.

**Abstract:**

Despite early detection and regular surveillance of non-muscle invasive bladder cancer (NMIBC), recurrence and progression rates remain exceedingly high for this highly prevalent malignancy. Limited visualization of malignant lesions with standard cystoscopy and associated false-negative biopsy rates have been the driving force for investigating alternative and adjunctive technologies for improved cystoscopy. The aim of our systematic review and meta-analysis was to compare the sensitivity, specificity, and oncologic outcomes of photodynamic diagnosis (PDD) fluorescence, narrow band imaging (NBI), and conventional white light cystoscopy (WLC) in detecting NMIBC. Out of 1,087 studies reviewed, 17 prospective non-randomized and randomized controlled trials met inclusion criteria for the study. We demonstrated that tumor resection with either PDD and NBI exhibited lower recurrence rates and greater diagnostic sensitivity compared to WLC alone. NBI demonstrated superior disease sensitivity and specificity as compared to WLC and an overall greater hierarchical summary receiver operative characteristic. Our findings are consistent with emerging guidelines and underscore the value of integrating these enhanced technologies as a part of the standard care for patients with suspected or confirmed NMIBC.

## 1. Introduction

Bladder cancer (BCa) represents one of the most common malignancies diagnosed in both males and females with a projected 2021 incidence of 83,730 and mortality of 17,200 in the United States [1]. Approximately 70% of BCa diagnoses present with non-muscle-invasive BCa (NMIBC): Ta, T1, and carcinoma in situ (CIS) [2]. Although BCa is typically detected in the early stages, there are significant five-year recurrence and progression rates of 78% and 45%, respectively [3]. Given the high risk of both recurrence and progression, regular cystoscopic surveillance is considered the standard of care following the first transurethral resection of a bladder tumor (TURBT) [4]. Although TURBT with conventional white light cystoscopy (WLC) is the prevailing method for detecting urothelial tumors [4], WLC has a false-negative rate of 10–20% due to limited lesion visualization [5]. As a result, novel technologies are under development to improve lesion detection, diagnostic accuracy, and prognosis.

The so-called “blue light cystoscopy”, also known as photodynamic diagnosis (PDD) fluorescence cystoscopy, was first described in 1964 [6] and due to its effectiveness in improving cancer detection and clinical outcomes, it has since gained popularity as an adjunct to WLC [7,8]. PDD is preceded by intravesical instillation of 5-aminolaevulinic acid (5-ALA) or hexaminolevulinate (HAL), photosensitizing prodrugs that preferentially induce the accumulation of porphyrins, most notably protoporphyrin IX, in rapidly proliferating urothelial cells. Illumination of the bladder wall with blue light (380–450 nm) causes cells with accumulated protoporphyrin to turn fluoresce red, aiding in neoplasm identification. Another imaging technique known as narrow band imaging (NBI) involves illuminating the bladder wall with filtered white light. The emitted wavelengths are absorbed by hemoglobin and thus penetrate the urothelial surface and enhance the visualization of the mucosal vasculature, especially neoangiogenic urothelial tumors [4]. While several clinical trials have been published examining the clinical outcomes associated with NBI, PDD, and WLC, there are no meta-analyses comparing the utility and outcomes of all three modalities.

In this study, we present a comprehensive systematic review and diagnostic meta-analysis comparing the diagnostic accuracy of WLC, NBI, and PDD in patients with BCa.

## 2. Materials and Methods

This study was performed following guidelines set out by the PRISMA (Preferred Reporting Items for Systematic Reviews and Meta-analysis) statement [9]. The study is registered in PROSPERO (CRD42017069333).

### 2.1. Search Strategy and Selection Criteria

We identified English-only prospective clinical trials of NBI, PDD, and WLC in bladder cancer through a search of PubMed/MEDLINE, Scopus, and Web of Science with the terms *“*5- *aminolevulinate *(or *5-ALA) blue-light cystoscopy”* OR “ *Hexaminolevulinate (* or *HAL) blue-light cystoscopy”* OR *“Narrow band imaging cystoscopy”* AND *“bladder cancer”* (Appendix A). We included trials published June 2021 or earlier that report diagnostic outcomes comparing 5-*aminolevulinate (5-ALA) blue-light cystoscopy* OR *Hexaminolevulinate (HAL) blue-light cystoscopy* OR *Narrow Band Imaging (NBI) cystoscopy* vs. *white light cystoscopy (WLC)*. Eligible studies were divided into 3 categories: (1) prospective clinical trials comparing 5-ALA vs. WLC; (2) prospective clinical trials comparing HAL vs. WLC; and (3) prospective clinical trials comparing NBI vs. WLC. Papers not reporting the diagnostic accuracy of the techniques were excluded from the meta-analysis. Editorials, commentaries, meeting abstracts, reviews, meta-analyses, book chapters, and studies reporting experiments involving human cadavers or animals were excluded from this review. References were manually reviewed to identify additional studies of interest.

### 2.2. Selection Studies and Quality Assessment

Two of the study authors (GEC and GIR) independently reviewed the literature according to the previously described inclusion and exclusion criteria. All discrepancies in study inclusion or exclusion were jointly reviewed until agreement was reached on the full list of articles. In instances where an institution or group published multiple papers derived from analyses of a single dataset, we carefully avoided duplicate data and extracted the most up-to-date parameters and endpoints of interest. Similarly, multi-site studies were excluded if the data and results overlapped with an included publication from a contributing center.

Following the Cochrane Handbook for Systematic Reviews, the risk of bias and study applicability were assessed using the validated Quality Assessment of Diagnostic Accuracy Studies (QUADAS-2) scoring system [10] and the Cochrane Collaboration’s Tool. Quality assessment of the studies was independently performed by two reviewers (GEC and GIR).

All papers were classified according to their level of evidence (LOE) for therapeutic studies: systematic review of randomized trials or *n*-of-1 trials (level 1); randomized trials or observational studies with dramatic effect (level 2); non-randomized controlled cohort or follow-up studies (level 3); case series, case-control studies, or historically-controlled studies (level 4); and mechanism-based reasoning (level 5).

### 2.3. Endpoints of Interests

The primary endpoint of interest was the per-lesion diagnostic accuracy of NBI vs. WLC and PDD vs. WLC. This included the cumulative tumor detection rate and false-positive rate stratified as appropriate for each diagnostic approach. These calculations were performed on lesion-level data.

### 2.4. Statistical Analysis

The cumulative meta-analysis of trials comparing NBI vs. WLC, 5-ALA vs. WLC, and HAL vs. WLC was conducted using Review Manager^®^ 5.3 (Cochrane Collaboration, Oxford, UK). We performed a comparative baseline characteristics analysis to evaluate statistically significant differences between patients who underwent 5-ALA, HAL, NBI, or WLC. The sensitivity analysis comparing HAL vs. WLC was carried out. All results were reported with 95% confidential intervals.

As part of the primary endpoint of the meta-analysis, we generated forest plots to assess result variability and heterogeneity, and then generated a receiver operating characteristic (ROC) curve to assess sensitivity and specificity. The ln(OR) and SE[ln(OR)] were calculated through a first-order Taylor series conversion, where SE[ln(OR)] = (1/OR) *SE[OR].

Primary outcomes are presented as pooled estimates of sensitivity and specificity with 95% confidence intervals (CIs) for detecting bladder cancer. Random or fixed effect were used in the case or absence of heterogeneity, respectively. To provide this result, we used the “metandi” command in Stata v.12.1 (StataCorp, College Station, TX, USA). The summary receiver operating characteristic (ROC) curve was plotted using this procedure. The pooled estimates for sensitivity and specificity were based on bivariate analysis.

## 3. Results

### 3.1. Literature Search

**Figure 1:**
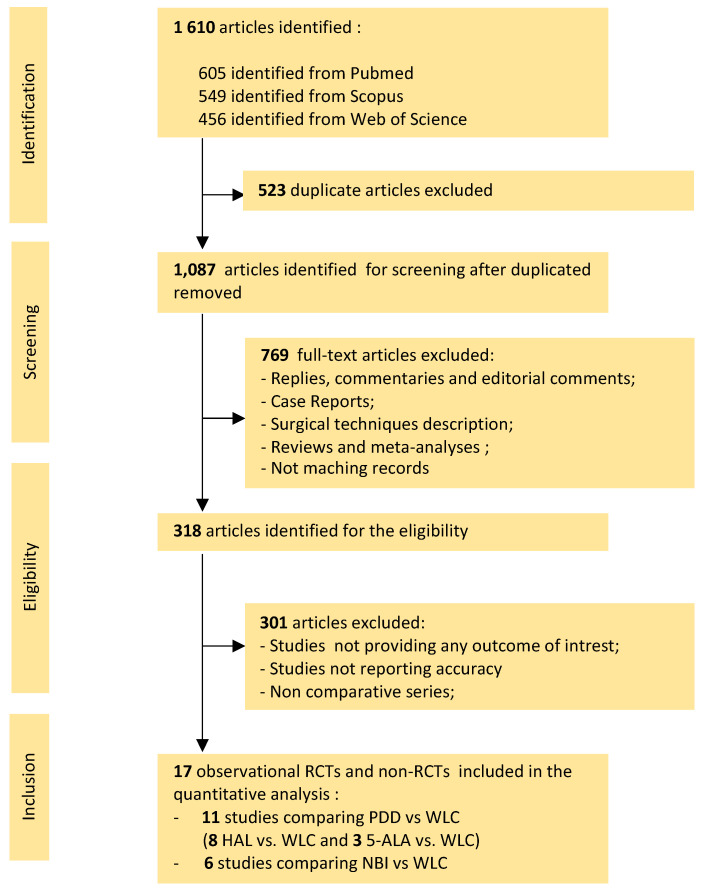
Study flowchart in accordance with the PRISMA statement.

Our initial systematic literature search yielded 1610 articles, 523 of which were duplicate studies. Screening of the remaining 1087 titles and abstracts generated 318 potentially eligible original articles. After careful review, 17 studies were retrieved and included in the quantitative analysis. Table 1 includes six studies comparing NBI vs. WLC [11,12,13,14,15,16]: one RCT (LOE 2) and five prospective non-RCTs (LOE 3). Table 2 includes three studies comparing 5-ALA vs. WLC [17,18,19], of which none were RCTs (LOE 2) and three were prospective non-RCTs (LOE 3), as well as eight studies comparing HAL vs. WLC [20,21,22,23,24,25,26,27], of which three were RCTs (LOE 2) and six were prospective non-RCTs (LOE 3). The study selection process is summarized in Figure 1.

### 3.2. Quality Assessment

**Figure 2:**
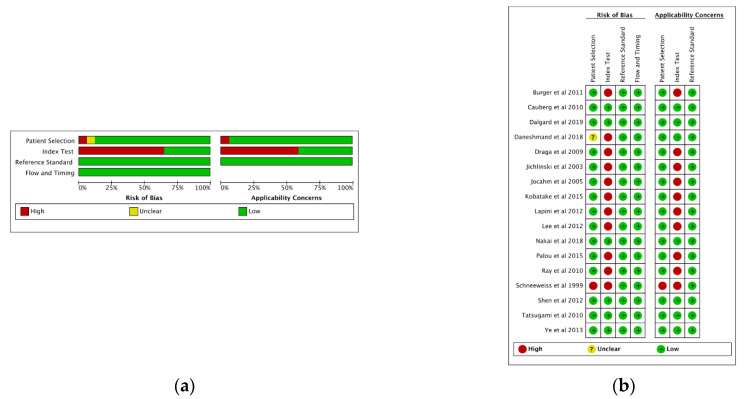
(**a**) Risk of bias and applicability-related concerns and (**b**) risk of bias and applicability concerns summary: review authors’ judgments about each domain for the included studies.

The overall quality of the studies is reported in Figure 2. None of the individual studies explicitly followed the Standards for Reporting of Diagnostic Accuracy (STARD) guidelines. All studies were analyzed according to the QUADAS-2 criteria. Within the patient selection domain, only one study had a high risk of bias due to inappropriate selection criteria [19]. All studies included were prospective. In the majority of studies, there was a high risk of bias in the index test domain due to the knowledge of results, potentially influencing the interpretation of the results. Within the reference standard and the flow and timing domains, all studies were determined to have a low risk of bias. The overall median QUADAS-2 score was 10.0 (range: 8.0–14.0).

### 3.3. Cumulative Accuracy Meta-Analysis of Comparative Studies Reporting Diagnostic Accuracy

**Figure 3:**
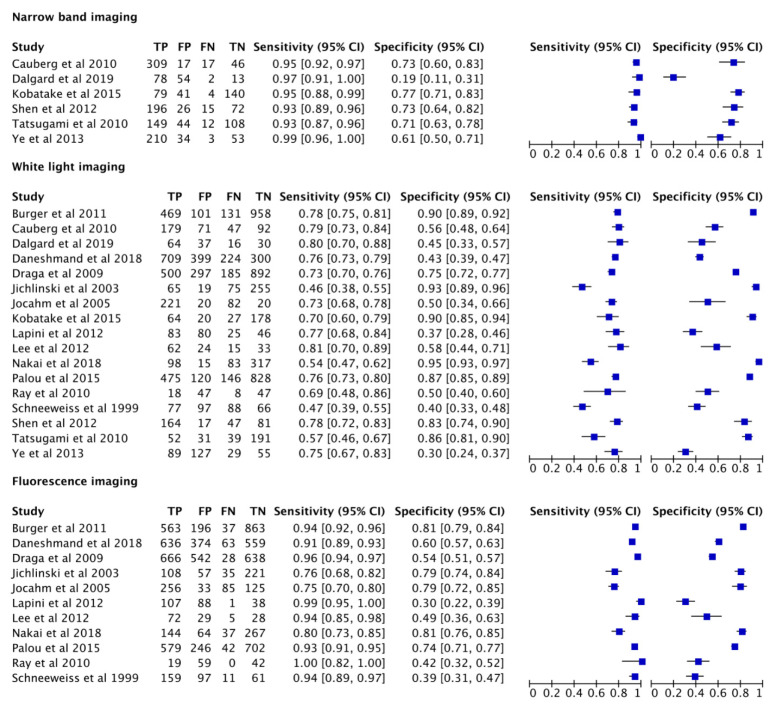
Forest plots of pooled specificity and sensitivity for all studies investigating NBI, white light imaging, and PDD for overall bladder cancer detection. Horizontal lines indicate 95% confidence intervals.

**Figure 4:**
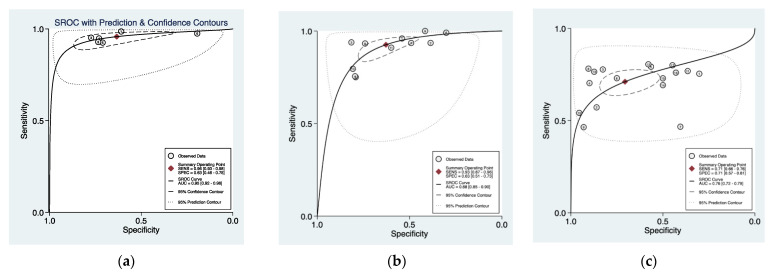
Hierarchical summary receiver operating characteristic (HSROC) plot (solid line) and summary point with a 95% confidence interval (circled area) of (**a**) NBI, (**b**) PDD, and (**c**) white light imaging in detecting overall bladder cancer. The dashed line represents the line of no discrimination (area under the curve of 0.5). Squares denote data from individual studies included in the meta-analysis with the size of each square indicating the relative size of the study population.

The pooled data showed a sensitivity of 0.96 (95% CI = 0.93–0.98), 0.93 (95% CI = 0.87–0.96), and 0.71 (95% CI = 0.66–0.76), and a pooled specificity of 0.65 (95% CI = 0.54–0.75), 0.63 (95% CI = 0.51–0.73), and 0.71 (95% CI = 0.57–0.81) for NBI, PDD, and WLC, respectively (Figure 3)**.** The derived area under the curve (AUC) from the hierarchical summary receiver operating characteristic (HSROC) showed an accuracy of 0.90 (95% CI = 0.92–0.98), 0.88 (95% CI = 0.85–0.90), and 0.76 (95% CI = 0.72–0.79) for NBI, PDD, and WLC, respectively (Figure 4).

Chi-square evaluation of the variation due to study heterogeneity was as follows: NBI I^2^ 93.0% (95% CI = 87.0–99.0; *p* < 0.01), 99% (95% CI = 99.0–100.0; *p* < 0.01), and 100% (95% CI = 100.0–100.0; *p* < 0.01).

The diagnostic OR for NBI, PDD, and WLC were 39.0 (95% CI = 24.0–64.0), 21.0 (95% CI = 14.0–32.0), and 6.0 (95% CI = 3.0–10.0), respectively.

The positive and negative likelihood ratio were 2.6 (95% CI = 1.7–4.0) and 0.07 (95% CI = 0.04–0.10] for NBI; 2.5 (95% CI = 1.9–3.3) and 0.12 (95% CI = 0.08–0.19) for PDD; and 2.4 (95% CI = 1.6–3.6) and 0.41 (95% CI = 0.34–0.50) for WLC.

**Figure 5:**
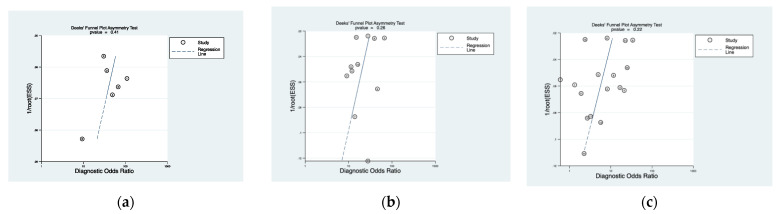
Funnel plots of Deeks et al. for (**a**) NBI, (**b**) PDD, and (**c**) WLC.

Figure 5 shows the funnel plot of Deeks et al. [28], demonstrating low risk of bias for NBI (*p* = 0.41), PDD (*p* = 0.26), and WLC (*p* = 0.22).

### 3.4. Subset Diagnostic Meta-Analysis of Studies Comparing HAL-PDD vs. WLC

As HAL-PDD have shown superiority over 5-ALA-PDD, we performed a sensitivity analysis including a total of eight studies that investigated the diagnostic accuracy of HAL-PDD vs. WLC.

**Figure 6:**
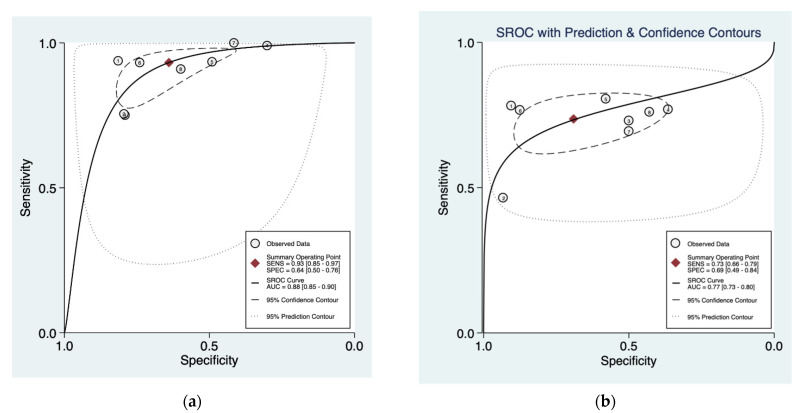
Hierarchical summary receiver operating characteristic (HSROC) plot (solid line) and summary point with a 95% confidence interval (circled area) of (**a**) PDD (HAL) and (**b**) white light imaging in detecting overall bladder cancer. The dashed line represents the line of no discrimination (area under the curve of 0.5). Squares denote data from individual studies included in the meta-analysis with the size of each square indicating the relative size of the study population.

The pooled data showed a sensitivity of 0.93 (95% CI = 0.85–0.97) and 0.73 (95% CI = 0.66–0.79), and a pooled specificity of 0.64 (95% CI = 0.50–0.76) and 69 (95% CI = 0.49–0.84) for HAL-PDD and WLC, respectively (Figure 6). The derived area under the curve (AUC) from the hierarchical summary receiver operating characteristic (HSROC) showed an accuracy of 0.88 (95% CI = 0.85–0.90) and 0.77 (95% CI = 0.73–0.80) for HAL-PDD and WLC, respectively (Figure 6).

The diagnostic OR for HAL-PDD and WLC were 24.0 (95% CI = 14.0–44.0) and 6.0 (95% CI = 3.0–13.0), respectively, while the positive and negative likelihood ratios were 2.6(95% CI = 1.9- 3.6) and 0.11 (95% CI = 0.06–0.20] for HAL-PDD and 2.4 (95% CI = 1.4–4.1) and 0.39 (95% CI = 0.29–0.51) for WLC.

## 4. Discussion

The cornerstone of diagnosis and surveillance of NMIBC is thorough cystoscopic bladder examination with histological examination of biopsies or resected tissue. While the use of WLC alone can lead to inaccurate diagnoses due to the limited visualization of neoplastic lesions [29], the role of PDD or NBI in increasing the accuracy of WLC and NMIBC management is still being explored. Despite evidence of a statistically significant improvement in the detection of primary and recurrent BCa with PDD and NBI, concerns have been raised regarding potential biases. These biases include observer bias, lack of blinding, and, in cases where NBI or PDD followed WLC with the same urologist, increased detection rate due to ‘‘second look” inspection of the bladder [4,13].

In the present study, we found that WLC was inferior to PDD and NBI in terms of both diagnostic OR and sensitivity. Based on the direct comparison, there were increased diagnostic ORs and sensitivities of NBI and PDD vs. WLC. The average sensitivity of NBI and PDD was 0.96 (0.93–0.98) and 0.93 (0.87–0.96), respectively, while that of WLC was 0.71 (0.66–0.76). The average diagnostic OR was 39 (24.0–64.0) and 21.0 (14.0–32.0) for NBI and PDD, respectively, and that of WLC was 6.0 (95% CI = 3.0–10.0). When the analysis was restricted to the studies comparing HAL-PDD vs. WLC only, the HAL-PDD cystoscopies were more sensitive than WLC (0.93 (95% CI = 0.85–0.97) vs. 0.73 (95% CI = 0.66–0.79)) with a higher diagnostic OR (24.0 (95% CI = 14.0–44.0) vs. 6.0 (95% CI = 3.0–13.0)).

A previous network intervention-meta-analysis of outcomes for TUR with PDD vs. NBI showed that the recurrence rate of cancers resected with 5-ALA-based PDD was significantly lower than those resected using HAL-based PDD (OR = 0.48 [95% CI = 0.26–0.95]) but was not significantly different than those resected with NBI (OR = 0.53 [95% CI = 0.26–1.09]). Similarly, the difference in the recurrence rate of cancers resected using HAL-based PDD vs. NBI was not statistically significant (OR = 1.11 [95% CI = 0.55–2.1]). Overall, NMIBC lesions resected with 5-ALA-based PDD, HAL-based PDD, and NBI recurred at a lower rate than those resected using WLC. Yet, no statistically significant difference in the progression rate was appreciated between cancers resected by all the methods investigated [30].

Consistent with our results, a previous meta-analysis by Xiong et al. showed that in a per-lesion analysis, the pooled additional detection rate of NBI for NMIBC was 18.6% greater than WLC [31]. The per-patient pooled sensitivity of NBI was significantly greater than WLC (95.8% vs. 81.6%, respectively) [31]. Furthermore, NBI significantly reduced the recurrence rate of bladder cancer with a pooled RR value of 0.43 (95% CI = 0.23–0.79) and 0.81 (95% CI = 0.69–0.95) at three- and twelve-months post-resection, respectively [31].

Chen et al. recently published a meta-analysis of observational studies assessing the diagnostic performance of NBI, 5-ALA, and HAL, concluding that the mage technique based transurethral resection (NBI, HAL, and 5-ALA) showed a diagnostic advantage [32]. In our systematic review, we have performed a “per lesion level meta-analysis’ of only prospective studies and provided the reference with the WLC.

However, it is worth noting that individual studies of the utility of NBI as an adjunct to WLC are quite variable. A single-center, randomized, and non-blinded study comparing same-session second-look with NBI and WLC found that although NBI detected a significantly greater number of lesions than WLC alone (*p* = 0.035), there was no statistically significant difference in recurrence (*p* = 0.373) or overall tumor detection (*p* = 0.137) [33]. The forthcoming Cochrane review of RCTs involving TURBT with NBI vs. WLC by Lai et al. may offer clarity concerning long-term clinical and oncologic outcomes [34].

The improved accuracy of NBI over WLC can be attributed to differences in the associated wavelengths of light. The spectrum of light used in NBI is relatively narrow (from 415 nm ultraviolet to 540 nm green) when compared to WLC, which includes a wide, non-standardized spectrum of light. In NBI, the visualization of microvascular structures is enhanced as the blue to green wavelengths penetrate superficial layers of the mucosa [35]. Typical limitations of NBI, such as blood altering light penetration and a procedural learning curve, do not seem to play a role in the cystoscopy for bladder cancer [36].

In comparison, PDD requires the instillation of prodrugs that, due to differences in enzymatic activity between malignant and benign tissues, lead to a selective accumulation of the fluorescent protoporphyrin IX in dysplastic cells [37,38]. The two most common agents used for PDD are 5-ALA and HAL, prodrugs that exhibit no photoactivity until they are metabolized in urothelial cells. In line with this mechanism, an analysis of a prospective US registry of PDD with HAL in combination with WLC revealed an increase in the detection of clinically relevant tumors with an impact on management [27]. Moreover, patient smoking status has not been found to impact recurrence rates in patients followed with PDD, though evidence of the impact of smoking on recurrence with WLC surveillance is equivocal [39]. A phase III prospective study of PDD with HAL found that approximately 20.6% of malignant lesions were only detected with PDD and not WLC, leading to a US consensus statement recommending PDD with HAL for surveillance cystoscopy of NMIBC [8,40]. Adherence to this consensus recommendation for NMIBC surveillance has shown that the use of PDD led to the detection of 33% cancerous lesions over WLC alone [41].

In addition to clinical outcomes, we also believe it is essential to consider the economic impact of increasingly complex technology. Although PDD has a higher upfront cost, previous studies have found that, due to improved tumor detection and more complete resection, PDD is more cost-effective than WLC in the long term [37,42]. Even in the short term, Smith et al. found that patients were willing to pay out-of-pocket despite the increased cost of PDD and demonstrated positive perceptions of PDD regardless of oncologic outcomes [43]. Sievert et al. found that the TURB cost for conventional WLC was EUR 1527.11 (USD 2031.06), while PDD had a cost of EUR 1386.83 (USD 1844.48) with a net cost saving of EUR 140.28 (USD 168.57) in a German health system [44]. Although not formally reviewed in our study, we would predict that the cost comparison of NBI to WLC would yield a similar result. The direct fixed equipment costs of NBI and PDD are likely comparable, though NBI would be expected to have lower direct variable costs as it does not require pre-procedural catheters and drug instillation.

Considering the findings of the present study, as well as the comparable publications and economic modeling, it is reasonable to recommend cystoscopy with either PDD or NBI for TURBT and surveillance of NMIBC. Currently, both the European Association of Urology (EAU) and the American Urological Association (AUA) recommend performing PDD cystoscopy/TURB (Grade B) to increase detection and recurrent lesions [4,45]. At this time, only the AUA guidelines recommend consideration of NBI (Grade C) to increase detection and decrease recurrence of NMIBC [4,45].

A 2019 survey assessing discordance between EAU guidelines for the management of NMIBC and clinical practice found that although a majority of European physicians endorse guideline adherence, a minority of patients receive PDD [46]. In patients who receive treatment for NMIBC in one of the surveyed European countries, the portion of patients undergoing PDD-TURB ranged from 1% to 40% (mean 15%) in low-risk BCa and from 4% to 55% (mean 28%) in high-risk BCa [46]. The relatively low and simultaneously heterogeneous adherence to EAU guidelines highlights a putative difference in the health policy between these countries and areas for future improvement.

While the primary strength of the present study is the inclusion of only prospective and randomized clinical studies, there several limitations exist. Firstly, the overall quality of the diagnostic studies was moderate and variable (median 10.0; range of 4 to 14), demonstrating the presence of bias in the included studies. We believe that observer and lack of blinding in the included studies may represent the most significant potential source of bias in the present study. Secondly, we made an intentional decision to conduct this meta-analysis at a per-lesion level rather than on a per-patient basis in order to increase the precision of sensitivity and specificity calculations for each diagnostic procedure and in the attempt to reduce the heterogeneity between the studies’ settings. Thirdly, cystoscopy is dependent on operator experience, which could not be accounted for in this study due to a lack of data. Finally, we have performed a meta-analysis of diagnostic test accuracy studies, which differs from the previous meta-analysis of therapeutic/interventional studies in which it was required to simultaneously analyze a pair of two outcome measures such as sensitivity and specificity rather than of a single outcome. It is worthy to note that the meta-analysis of diagnostic test accuracy studies is a more sensitive tool when comparing different diagnostic tests.

Given our findings we believe that ongoing interventional trials will continue to strengthen evidence supporting adjunct technologies for NMIBC diagnosis, surveillance, and management. Future research is needed to directly evaluate differences in the clinical outcomes and economic burden between PDD and NBI. We anticipate the development of novel techniques based on PDD and NBI. In PDD, there is an ongoing pilot study (NCT03058705) assessing a highly sensitive multi-spectral imaging modality (near infrared fluorescence or NIRF) with the potential to speed up the detection of bladder cancer fluorescence after the infusion of hexaminolevulinate compared to the standard PDD. Both PDD and NBI are also being evaluated for their potential to evaluate surgical margins intraoperatively and thereby improve long-term outcomes for patients with NMIBC [36,47].

## 5. Conclusions

In this meta-analysis, we demonstrated that TURBT with either PDD or NBI exhibited a greater diagnostic sensitivity compared to WLC. Our findings underscore the value of integrating these enhanced technologies as a part of the standard care for patients with suspected or confirmed NMIBC.

## Figures and Tables

**Table 1:** Studies comparing narrowband imaging vs. white light cystoscopy. Abbreviations: RTC, randomized clinical trial; LOE, level of evidence; NBI, narrowband imaging; and WLC, white light cystoscopy. The data reported are per patient-level.

Study	Year	Institution	Type of Study	LOE	Type of Cystoscopy	Number of Samples
Ye et al. [16]	2013	Huazhong University of Science and Technology, Wuhan, China	RTC	2	NBI	300
					WLC	300
Tatsugami et al. [15]	2010	Kyushu University, Fukuoka, Japan	Prospective	3	NBI	313
					WLC	313
Song et al. [14]	2014	Department of Urology, Yeungnam University College of Medicine, Daeg, Korea	Prospective	3	NBI	63
					WLC	63
Shen et al. [13]	2012	Department of Urology, Fudan University Shanghai Cancer Center	Prospective	3	NBI	309
					WLC	309
Kobatake et al. [12]	2015	Department of Urology, Hiroshima City Asa Hospital, Hiroshima 731-0293, Japan	Prospective	3	NBI	264
					WLC	289
Cauberg et al. [11]	2010	Departments of Urology and Pathology, Medical Center, Amsterdam	Prospective	3	NBI	389
					WLC	389

**Table 2:** Studies comparing photodynamic diagnosis vs. white light cystoscopy. Abbreviations: RTC, randomized clinical trial; LOE, level of evidence; 5- ALA, 5-aminolevulinic acid; HAL, hexaminolevulinate; and WLC, white light cystoscopy. The data reported are per patient-level.

Study	Year	Institution	Type of Study	LOE	Type of Cystoscopy	Number of Samples
Draga et al. [17]	2009	Departments of Urology, Medical Physics, and Pathology, University Medical Center Utrecht, Utrecht, the Netherlands Medical Center Utrecht	Prospective	3	5-ALA	1874
					WLC	1874
Schneeweiss et al. [19]	1999	Department of Epidemiology, Harvard University School of Public Health, Boston, Massachusetts	Prospective	3	5-ALA	328
					WLC	328
Burgues et al. [20]	2011	Department of Urology, Caritas-St. Josef Medical Center, University of Regensburg, Regensburg, Germany	RCT	2	HAL	1659
					WLC	1659
Nakai et al. [18]	2018	Department of Urology, Nara Medical University, Japan	Prospective	3	5-ALA	61
					WLC	61
Jichlinski et al. [21]	2003	Department of Urology and Institute of Pathology, CHUV University-Hospital	Prospective	3	HAL	421
					WLC	414
Jocahm et al. [22]	2005	Departments of Urology, University of Schleswig-Holstein, Campus Lübeck (DJ), Lübeck	RCT	2	HAL	499
					WLC	343
Lapini et al. [23]	2012	Department of Urology (PJ, H-JL) and Institute of Pathology (LG), CHUV University-Hospital	Prospective	3	HAL	234
					WLC	234
Lee et al. [24]	2012	Department of Urology, Samsung Medical Center, Sungkyunkwan University School of Medicine, Seoul	Prospective	3	HAL	110
					WLC	134
Palou et al. [25]	2015	Fundacio Puigvert, Universitat Auto noma de Barcelona, Barcelona	Prospective	3	HAL	1569
					WLC	1569
Ray et al. [26]	2010	Urology Centre, Guy and St. Thomas’ NHS Foundation Trust, London, UK	Prospective	3	HAL	120
					WLC	120
Daneshmand et al. [27]	2018	US prospective multicenter registry	Prospective	3	HAL	1632
					WLC	1632

## Data Availability

The data presented in this study are available on request from the corresponding author.

## Appendix A

### Appendix A.1. Search Criteria and Translational Terms Used for the Literature Review

Search:(((((narrow band imaging) AND (bladder cancer)) OR ((NBI) AND (bladder cancer))) OR ((((Hexaminolevulinate) AND (bladder cancer)) OR ((5-aminolevulinate) AND (bladder cancer))) OR ((blue light cystoscopy) AND (bladder cancer)))) OR ((5-ALA) AND (bladder cancer))) OR ((HAL) AND (bladder cancer))

((“narrow band imaging” [MeSH Terms] OR (“narrow” [All Fields] AND “band” [All Fields] AND “imaging” [All Fields]) OR “narrow band imaging” [All Fields]) AND (“urinary bladder neoplasms” [MeSH Terms] OR (“urinary” [All Fields] AND “bladder” [All Fields] AND “neoplasms” [All Fields]) OR “urinary bladder neoplasms” [All Fields] OR (“bladder” [All Fields] AND “cancer” [All Fields]) OR “bladder cancer” [All Fields])) OR (“NBI” [All Fields] AND (“urinary bladder neoplasms” [MeSH Terms] OR (“urinary” [All Fields] AND “bladder” [All Fields] AND “neoplasms” [All Fields]) OR “urinary bladder neoplasms” [All Fields] OR (“bladder” [All Fields] AND “cancer” [All Fields]) OR “bladder cancer” [All Fields])) OR (((“5 aminolevulinic acid hexyl ester” [Supplementary Concept] OR “5 aminolevulinic acid hexyl ester” [All Fields] OR “hexaminolevulinate” [All Fields]) AND (“urinary bladder neoplasms” [MeSH Terms] OR (“urinary” [All Fields] AND “bladder” [All Fields] AND “neoplasms” [All Fields]) OR “urinary bladder neoplasms” [All Fields] OR (“bladder” [All Fields] AND “cancer” [All Fields]) OR “bladder cancer” [All Fields])) OR ((“5 aminolaevulinate” [All Fields] OR “aminolevulinic acid” [MeSH Terms] OR (“aminolevulinic” [All Fields] AND “acid” [All Fields]) OR “aminolevulinic acid” [All Fields] OR “5 aminolevulinate” [All Fields]) AND (“urinary bladder neoplasms” [MeSH Terms] OR (“urinary” [All Fields] AND “bladder” [All Fields] AND “neoplasms” [All Fields]) OR “urinary bladder neoplasms” [All Fields] OR (“bladder” [All Fields] AND “cancer” [All Fields]) OR “bladder cancer” [All Fields])) OR (“blue” [All Fields] AND (“light” [MeSH Terms] OR “light” [All Fields] OR “lighted” [All Fields] OR “lights” [All Fields] OR “lighting” [MeSH Terms] OR “lighting” [All Fields] OR “lightings” [All Fields] OR “lightness” [All Fields] OR “lightnesses” [All Fields]) AND (“cystoscopy” [MeSH Terms] OR “cystoscopy” [All Fields] OR “cystoscopies” [All Fields]) AND (“urinary bladder neoplasms” [MeSH Terms] OR (“urinary” [All Fields] AND “bladder” [All Fields] AND “neoplasms” [All Fields]) OR “urinary bladder neoplasms” [All Fields] OR (“bladder” [All Fields] AND “cancer” [All Fields]) OR “bladder cancer” [All Fields]))) OR ((“aminolevulinic acid” [MeSH Terms] OR (“aminolevulinic” [All Fields] AND “acid” [All Fields]) OR “aminolevulinic acid” [All Fields] OR “5 ala” [All Fields]) AND (“urinary bladder neoplasms” [MeSH Terms] OR (“urinary” [All Fields] AND “bladder” [All Fields] AND “neoplasms” [All Fields]) OR “urinary bladder neoplasms” [All Fields] OR (“bladder” [All Fields] AND “cancer” [All Fields]) OR “bladder cancer” [All Fields])) OR (“HAL” [All Fields] AND (“urinary bladder neoplasms” [MeSH Terms] OR (“urinary” [All Fields] AND “bladder” [All Fields] AND “neoplasms” [All Fields]) OR “urinary bladder neoplasms” [All Fields] OR (“bladder” [All Fields] AND “cancer” [All Fields]) OR “bladder cancer” [All Fields]))

### Appendix A.2. Translations

**narrow band imaging:** “narrow band imaging” [MeSH Terms] OR (“narrow” [All Fields] AND “band” [All Fields] AND “imaging” [All Fields]) OR “narrow band imaging” [All Fields]

**Hexaminolevulinate:**”5-aminolevulinic acid hexyl ester” [Supplementary Concept] OR “5-aminolevulinic acid hexyl ester” [All Fields] OR “hexaminolevulinate” [All Fields]

**5-aminolevulinate:**”5 aminolaevulinate” [All Fields] OR “aminolevulinic acid” [MeSH Terms] OR (“aminolevulinic” [All Fields] AND “acid” [All Fields]) OR “aminolevulinic acid” [All Fields] OR “5 aminolevulinate” [All Fields]

**light:**”light” [MeSH Terms] OR “light” [All Fields] OR “lighted” [All Fields] OR “lights” [All Fields] OR “lighting” [MeSH Terms] OR “lighting” [All Fields] OR “lightings” [All Fields] OR “lightness” [All Fields] OR “lightnesses” [All Fields]

**cystoscopy:**”cystoscopy” [MeSH Terms] OR “cystoscopy” [All Fields] OR “cystoscopies” [All Fields]

**5-ALA:**”aminolevulinic acid” [MeSH Terms] OR (“aminolevulinic” [All Fields] AND “acid” [All Fields]) OR “aminolevulinic acid” [All Fields] OR “5 ala” [All Fields]

**bladder cancer:**”urinary bladder neoplasms” [MeSH Terms] OR (“urinary” [All Fields] AND “bladder” [All Fields] AND “neoplasms” [All Fields]) OR “urinary bladder neoplasms” [All Fields] OR (“bladder” [All Fields] AND “cancer” [All Fields]) OR “bladder cancer” [All Fields]
